# The Effect of Glycosylation on Biodistribution and Cytotoxicity of ^99^mTc-Radiolabeled Luteolin

**DOI:** 10.34172/apb.025.46124

**Published:** 2025-10-21

**Authors:** Mostafa Pirali Hamedani, Mohammad Seyedhamzeh, Mohammad H. Ghahremani, Abbas Hadjiakhoondi, Morteza Pirali Hamedani, Mehdi Shafiee Ardestani, Zahra Tofighi

**Affiliations:** ^1^Department of Pharmacognosy and Pharmaceutical Biotechnology, Faculty of Pharmacy, Iran University of Medical Sciences, Tehran, Iran; ^2^Pharmaceutical Sciences Research Center, Medicinal Plants Research, Tehran University of Medical Sciences, Tehran, Iran; ^3^Department of Pharmaceutical Nanotechnology, Zanjan University of Medical Sciences, Zanjan, Iran; ^4^Department of Pharmacology and Toxicology, Faculty of Pharmacy, Tehran University of Medical Sciences, Tehran, Iran; ^5^Department of Medicinal Chemistry, Faculty of Pharmacy, Tehran University of Medical Sciences, Tehran, Iran; ^6^Department of Radiopharmacy, Faculty of Pharmacy, Tehran University of Medical Sciences, Tehran, Iran

**Keywords:** Skullcap, Radiolabeled flavonoids, SPECT imaging, MTT assay, Flow cytometry, Cell cycle arrest

## Abstract

**Introduction:**

Luteolin (Lu) and its glycosylated derivative, luteolin-7-O-glucoside (LuG), are the main bioactive flavonoids reported in the *Scutellaria* genus. Still, in vivo biodistribution and cytotoxic effects on cancer cells remain unrevealed. This study investigated the biodistribution and cytotoxic effects of Lu and LuG on cancer cell lines, particularly hepatic carcinoma (HepG2).

**Methods:**

Lu and LuG were isolated from *S. pinnatifida* extracts, and the structures were confirmed by ¹H-NMR spectroscopy. Radiolabeling was performed to assess their biodistribution in Wistar-Albino rats using SPECT imaging and organ radioactivity was measured. Organs were harvested and radioactivity quantified to determine tissue accumulation. Cytotoxicity was evaluated via MTT assay on normal (HUVEC) and cancer (HepG2, SW480) cell lines. Flow cytometry analyzed apoptosis/necrosis and cell cycle arrest after treatment.

**Results:**

LuG exhibited preferential accumulation in the liver (~28.7%) and significant cytotoxicity on HepG2 cells. Flow cytometry indicated non-apoptotic cell death and G1/S phase cell cycle arrest in HepG2 cells treated with LuG, whereas Lu showed less accumulation and cytotoxicity. Biodistribution data revealed lower accumulation in other organs, and LuG had negligible toxicity on non-hepatic cells (HUVEC, SW480). Overall biodistribution analysis revealed lower off-target accumulation, further supporting the hepatic selectivity of LuG.

**Conclusion:**

This study provides the first integrated evidence of the liver-targeted biodistribution and selective anticancer effects of LuG. These findings demonstrate LuG as a candidate therapeutic for hepatocellular carcinoma. These results emphasize the importance of pharmacological evaluation of flavonoid glycosides and support preclinical development of LuG as a targeted anticancer agent.

## Introduction

 Cancer arises from genetic and environmental factors leading to abnormal, uncontrolled cell growth.^1^ Numerous plants have demonstrated anticancer and antitumor effects, and studies have shown that phenolic compounds, especially flavonoids such as luteolin and its glycosylated derivatives, play a vital role in combating cancerous cells.^2,3^ luteolin and its derivatives have been found in many plants, including Apiaceae family (*Apium graveolens* L., *Daucus carota* L.), Lamiaceae family (*Scutellaria* spp.), and other families.^4^ The *Scutellaria* genus, a source of luteolin, is distributed worldwide, except in Antarctica.^5^ Among the approximately 350 species of *Scutellaria*, about 22 taxa are represented in Iran, with 10 of these species native to the Iranian Plateau in the Irano-Turanian region.^6^ Luteolin (3′,4′,5,7-tetrahydroxy flavone) is a member of the flavonoid super-group and belongs to the flavone class. Research has demonstrated that luteolin and its glycosylated variant possess a wide range of effects, including antioxidant, anti-inflammatory, antimicrobial, anti-diabetic, anti-hyperlipidemic, anticancer, and chemosensitizing properties.^7^ Previous studies showed that luteolin has anti-proliferative effects on various cancerous cell lines such as human prostate cancer (PC-3 cells), human non-small cell lung cancer (A549 cells), breast cancer (MCF-7 and MDA-MB-231 cells), human colon cancer (SW620 and HT-29 cells), and liver cancer (HepG2 cells), and can induce apoptosis pathway in these cell lines.^8,9^ Previous findings demonstrated that the IC_50_ of Luteolin (aglycone) was greater than that of the glycoside form of luteolin in specific cancer cell lines, such as breast cancer. Conversely, in other cancer cell lines, the IC_50_ of the glycoside form of luteolin is higher than that of the aglycone form.^10^ Several *in vivo* studies provide valuable insights into the actual biodistribution of flavonoids. It was observed that Tangeretin accumulates primarily in the kidneys, lungs, and liver, with lower levels found in the spleen and heart.^11^ B-ring unsubstituted flavones such as baicalein, wogonin, and oroxylin A, were distributed in the liver and kidney, with moderate levels in the prostate and low levels in the lungs and pancreas.^12^ However, a significant limitation is the lack of detailed data on flavonoid tissue distribution in humans. Moreover, *in vivo* studies assessing accumulation, metabolism, and retention in human tissues are exceedingly rare, despite their importance for understanding biological activity. This article investigates a hypothesis proposing a relationship between the bioaccumulation of luteolin and its glycoside form and their anti-proliferative effects. This study aims to compare the biodistribution of these forms of luteolin (aglycone and glycone) and their cytotoxic effects on the same organ in which they exhibit the highest accumulation.

## Materials and Methods

###  Plant Sample

 In 2020, *Scutellaria pinnatifida* was collected from Alamut road, Qazvin province, Iran. The identification of *S. pinnatifida* was conducted by botanist Dr. Y. Ajani, and the voucher specimen was registered with the 7040-TEH code in the Herbarium of the Faculty of Pharmacy, Tehran University of Medical Sciences, Tehran, Iran.

###  Isolated Luteolin and Luteolin-7-O-Glucoside

 According to a 2023 study by Pirali *et al*., Luteolin (Lu) and luteolin-7-*O*-glucoside (LuG) were isolated. The harvested plant was dried out of direct sun exposure at 25°C. A total of 230 g of powdered plant material was extracted with 80% methanol using the maceration method. After 48 h, the extract was filtered, concentrated, and this process was repeated three times. The weight of dry extract was about 30 g, resulting in a yield of about 13%. Approximately 10 g of dried extract was separated into phenolic and non-phenolic fractions using Diaion HP-20 resin. Three grams of the phenolic fraction were loaded onto a reversed-phase silica gel column and eluted with a gradient of water to methanol (8:2) to 100% methanol. Subsequently, the achieved sub-fractions were loaded onto Sephadex LH-20, and after several steps, luteolin and luteolin-7-*O*-glucoside were successfully isolated. The ¹H-NMR spectrum confirmed the structure of these isolated compounds.^4^

### ^99m^Tc Radio-Labeling of Luteolin and luteolin-7-O-Glucoside

 Five milligrams of tin chloride (SnCl_2_) was dissolved in 1 mL of hydrochloric acid 10% and diluted with 3 mL of distilled water, and the pH was adjusted to 7. Then, 1 mg of Lu and LuG and about 4 mCi of fresh pertechnetate (^99m^TcO_4_) in 2 mL of NaCl were added to the complex. Afterward, it was incubated at room temperature for 20 minutes.

###  Radiochemical Purity

 Radiochemical purity (RCP) was analyzed using Whatman grade No. 2 qualitative filter paper as the stationary phase and a methanol: normal saline (1:1) mixture as the mobile phase. The impurity of the radiochemical was evaluated by spotting six μL of the prepared complex at the bottom of a Whatman paper. The strips were divided into two parts, and the radioactivity of each part was determined using a gamma counter. When the mobile phase was acetone: methanol, the ^99m^TcO_2_-radiolabeled complex remains at the origin spot and ${}^{99m}TcO_{4}^{-}$ moved by the mobile phase. When the mobile phase was methanol: normal saline, ^99m^TcO_2_ remain at the original spot and ${}^{99m}TcO_{4}^{-}$ with radiolabeled complex was moved by mobile phase. Then, the percentage of ^99m^TcO_2_ was calculated, and the following formula assessed the RCPs:


$$Radiochemical\;purity=100-\sum_{count}{(}^{99m}TcO_{4}^{-}{+}^{99m}TcO_{2})$$

###  Biodistribution Studies

 An intraperitoneal injection of a ketamine and xylazine mixture anesthetized the Wistar-Albino rats. About 1.4 mCi of flavonoid-^99m^Tc complex (^99m^Tc-Lu and ^99m^Tc-LuG) was injected through the tail vein. The imaging was performed using SPECT (Single-Photon Emission Computed Tomography) at 0, 5, 15, 30, 60, and 90 min. Then, the animals were sacrificed in accordance with all ethical considerations, and each organ was carefully separated. The percentage of radioactivity in each organ was calculated by dividing the activity counted by the dose calibrator by the total radioactivity. This method ensured accurate assessment of the biodistribution of the radiolabeled compounds within the various organs.


$$\begin{array}{l} Organ\;accumulation\;of{\;}^{99m}Tc-Lu{/}^{99m}Tc-LuG\;(\%)=\\ (\frac{Amount\;of\;radioactivity\;counted\;in\;each\;organ}{Amount\;of\;radioactivity\;counted\;in\;whole\;body})\times 100 \end{array}$$

###  MTT Assay

####  Cell Culture

 According to the biodistribution results of Lu and LuG in the previous step, three cell lines were selected for further analysis. HUVEC cell line (an endothelial cell that was isolated from the umbilical cord vein) as a normal cell, SW480 as a colorectal cancer cell line, and HepG2 as a hepatocellular carcinoma cell line were obtained from the cell bank of Roshd Azma Company. They were cultured in Dulbecco’s Modified Eagle Medium (DMEM) supplemented with FBS 10% (Fetal Bovine Serum) and Pen/Sterp 10^4^ U/mL (Penicillin- Streptomycin) at 37°C and 5% carbon dioxide (CO_2_). Then, 10^5^ cells/mL were seeded in 96-well cell culture plates and incubated for 24 h.

####  Cell Viability Assay

 After 24 h, the cell lines were treated with Lu and LuG (5, 10, 25, 50, 75, 100, 200 µg/mL). The cytotoxicity effect of Lu and LuG was measured using the MTT (3-[4,5-dimethylthiazol-2-yl]- 2,5-diphenyltetrazolium bromide) assay for 24 h. Next, 20 μL of MTT solution (5 mg/mL) was added to each well, and the complexes were incubated at 37°C for 4 hours. Afterward, the culture medium was removed and replaced with DMSO 200 μL). The absorbance of each well was determined at 570 nm using an ELISA reader, and the cell viability percentage was calculated relative to the control group.

 The examination of cell viability percentage was repeated after 48 and 72 h for samples that exhibited higher cytotoxicity in their respective cancer cell lines.

###  Flow Cytometry

 According to the results of the previous steps, LuG was selected for flow cytometry testing on the HepG2 cancer cell line. FITC (fluorescin isothiocyanate) Annexin V, as a calcium-dependent membrane-binding protein, was bound to phosphatidylserine (PS), and Propidium Iodide (PI), as a fluorescent intercalating agent, was intercalated to DNA, which demonstrated the quantity of apoptosis and necrosis in cancer cells by FACScan flow cytometer. HepG2 cells were treated with LuG (5, 30, 50 µg/mL) for 48 h and then washed with PBS. Next, 300 µL of binding buffer was added to 1 × 10^6^ cells and incubated for 15 min at 25°C in away from the light with 4 µL of FITC Annexin V and 2 µL of PI. To investigate apoptotic or necrotic pathways, data were analyzed by FlowJo software. Data analysis revealed the differentiation between early apoptotic (Annexin V + /PI-), late apoptotic or necrotic (Annexin V + /PI + ), and necrotic cells (Annexin V-/PI + ). Additionally, cells without any treatment (negative control) and those treated with LuG (positive control) were designated as “Auto” and “Unstained”, respectively.

####  Cell Cycle Arrest

 HepG2 cells were seeded in 6-well plates and treated with various concentrations of Luteolin-7-*O*-glucoside for 24 h. After treatment, the cells were fixed in a solution of ethanol: water (7:3) at -20 °C. Then, ethanol-fixed cells were washed with PBS and incubated with RNase A (100 μL) for 30 min. Following the RNase treatment, the cells were incubated in a PI/Triton X-100 solution for an additional 30 minutes. The samples were analyzed using a flow cytometer (FACSCalibur), and the data were assessed with FlowJo version 10. Finally, the G0/G1 phase (2N DNA content), S phase (intermediate DNA content), and G2/M phase (4N DNA content) were calculated.^13^

## Results and Discussion

###  Identification of Isolated Compounds

**Figure 1:**
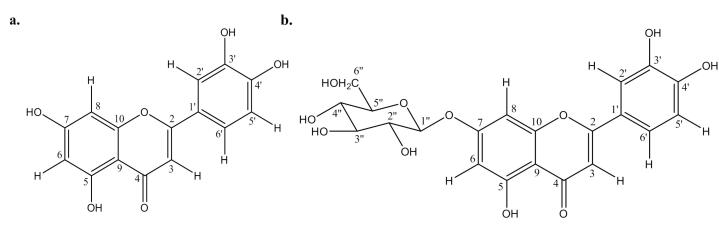
The structure of Luteolin and Luteolin-7-*O*-glucoside

 Two isolated compounds, through several chromatography steps, were identified, and the structures of Luteolin and Luteolin-7-*O*-glucoside were confirmed (Figure 1) using ¹H-NMR spectra (500 MHz, DMSO-d6), as shown in Table 1.

**Table 1:** ^1^H-NMR data of Luteolin and Luteolin-7-*O*-glucoside

	^1^**H-NMR spectra**	
**Carbon No.**	**Luteolin**	**Luteolin-7-** ***O*** **-glucoside**
1	-	-
2	-	-
3	6.60 (1H, *s*)	6.74 (1H, *s*)
4	-	-
5	-	-
6	6.13 (1H, *d*, *J* = 2 Hz)	6.45 (1H, *d*, *J* = 2 Hz)
7	-	-
8	6.39 (1H, *d*, *J* = 2 Hz)	6.80 (1H, *d*, *J* = 2 Hz)
9	-	-
10	-	-
1′	-	-
2′	7.36 (1H, *d*, *J* = 2 Hz)	7.43 (2H, *dd*, *J* = 8, 2 Hz)
3′	-	-
4′	-	-
5′	6.82 (1H, *d*, *J* = 8 Hz)	6.90 (2H, *d*, *J* = 8 Hz)
6′	7.40 (1H, *dd*, *J* = 8, 2 Hz)	7.43 (2H, *dd*, *J* = 8, 2 Hz)
1″		5.19 (1H, *d*, *J* = 7.5 Hz)
2″ to 6″		3.5-4.5 (hydrogens of glucose)

###  In-Vivo Biodistribution Study 

**Figure 2:**
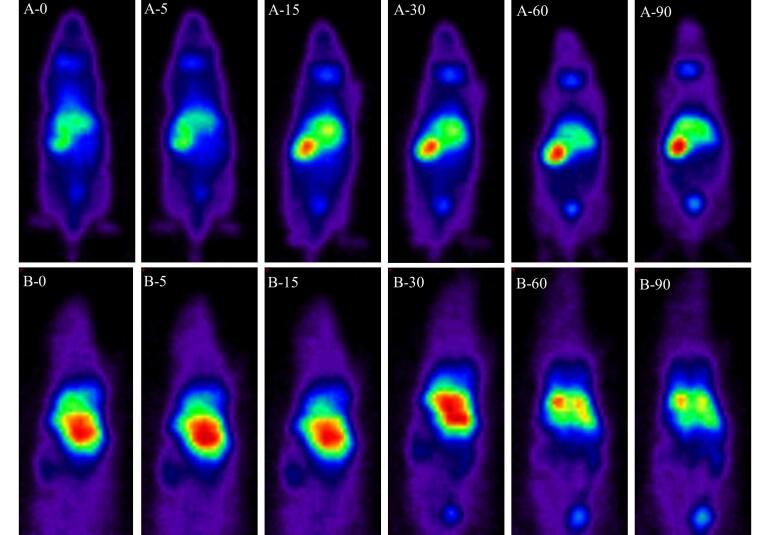
SPECT images of the biodistribution of ^99m^Tc-Luteolin (A) and ^99m^Tc-Luteolin-7-*O*-glucoside (B) in the whole body of the rat at 0, 5, 15, 30, 60, and 90 min after radioactive-flavonoid complex injection

^99m^Tc-Lu and ^99m^Tc-LuG were injected into rats, and whole-body SPECT images were acquired at various times (0, 5, 15, 30, 60, and 90 min) post-injection. These imaging sessions allowed for the visualization and assessment of the biodistribution and pharmacokinetics of the radiolabeled compounds over time, as illustrated in Figure 2. The percentage of biodistribution of ^99m^Tc-Lu and ^99m^Tc-LuG in each organ were summarized in Table 2. Higher accumulation of LuG in the liver than the aglycone form may be due to its different uptake mechanism. An experiment was conducted to elucidate carrier-mediated transports, such as SGLT1/GLUT1, Which Participate in the uptake and distinct absorption pathways for glycosides versus aglycones of flavonoids in Caco2 cells.^14^ Also, Hepatic uptake transporters like organic anion-transporting polypeptides (OATP1B1/1B3) preferentially accept certain glycosides. Flavonoid glycosides are better substrates for OATPs than aglycone flavonoids, promoting hepatic accumulation. Experimental studies show flavonoids interact with OATP1B1 and related uptake transporters.^15^

**Table 2:** The percentage ^99m^Tc-Lu and ^99m^Tc-LuG accumulation for each organ in animals

**Organ**	^99m^**Tc-Lu biodistribution (%)**	^99m^**Tc-LuG biodistribution (%)**
Liver	23.0	28.7
Stomach	13.4	28.7
Intestine	6.4	6.9
Bladder	2.4	6.0
Lung	0.5	5.6
Kidney	0.6	1.7
Spleen	0.1	0.5
Testis	0.1	0.1
Blood	0.0	0.1
Heart	0.0	0.2
Brain	0.0	0.0
Bone	0.0	0.0

###  Cell Viability

**Figure 3:**
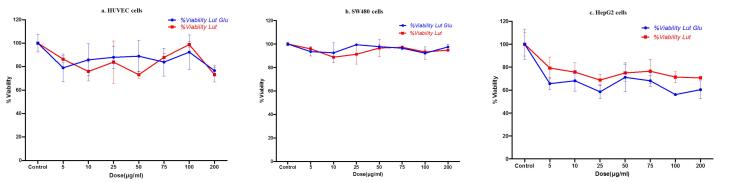
The cell viability percentage (mean ± SEM) of luteolin (red) and luteolin-7-*O*-glucoside (blue) with different concentrations by MTT assay. **a.** HUVEC, **b.** SW480, **c.** HepG2

 The effect of luteolin and luteolin-7-*O*-glucoside at various concentrations on the viability of HUVEC, SW480, and HepG2 cells after 24 h, as assessed by the MTT assay, is illustrated in Figure 3.

**Figure 4:**
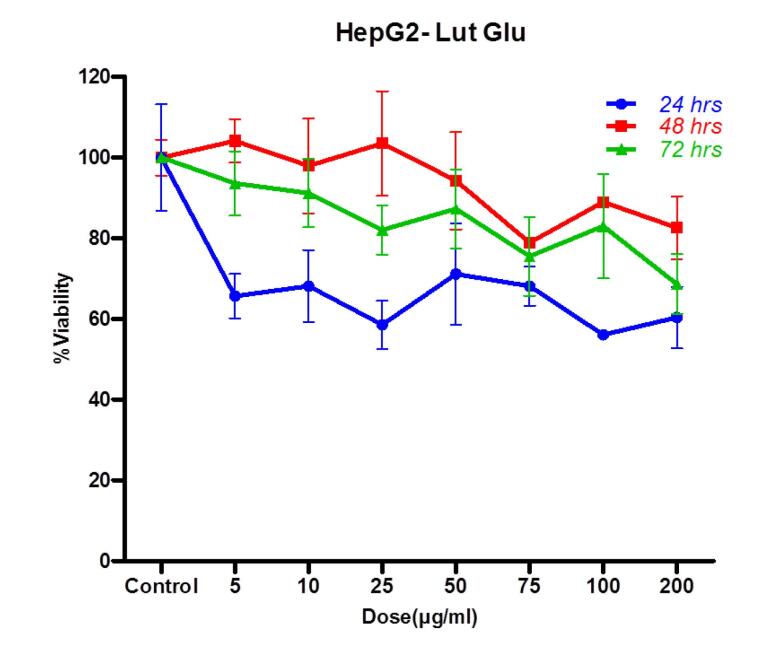
The percentage of HepG2 cells viability against different concentrations of luteolin-7-*O*-glucoside treated after 24 (blue), 48 (red), and 72 h. (green) by MTT assay (mean ± SEM)

 The results of the MTT assay indicated that luteolin-7-*O*-glucoside exhibited the highest cytotoxic effect on the HepG2 cancer cell line compared to the other cancer cell lines assessed. To further investigate, the cytotoxicity effect of this flavonoid was evaluated on HepG2 cells at 48 and 72 h. The findings regarding cell viability are demonstrated in Figure 4.

###  Flow cytometry data

**Figure 5:**
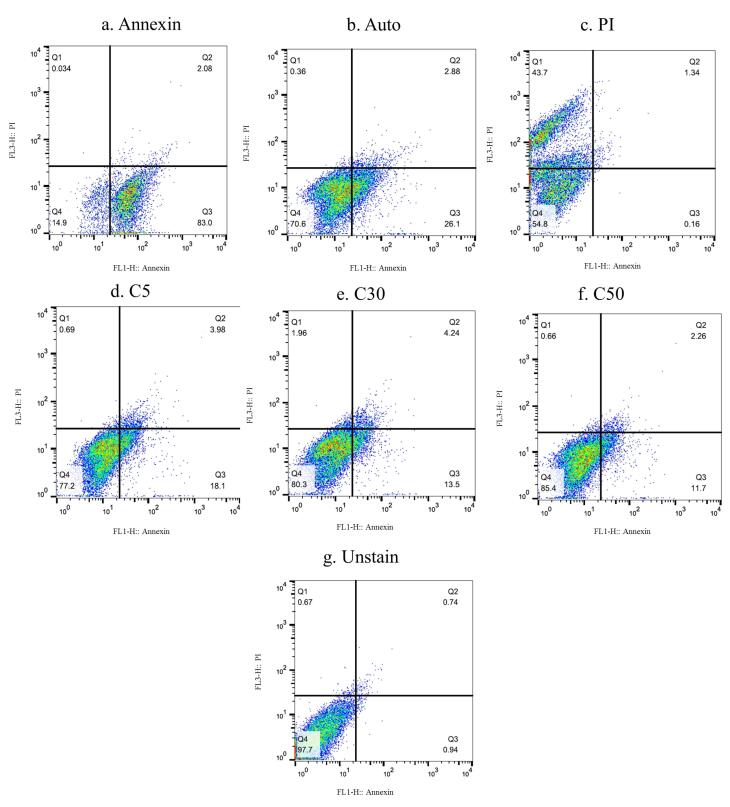
Double staining of Luteolin-7-*O*-glucoside with annexin and propidium iodide. **a.** Annexin group, **b.** Auto group, **c.** PI group, **d.** LuG at 5 µg/mL concentration, **e.** LuG at 30 µg/mL concentration, **f.** LuG at 50 µg/mL concentration, **g.** unstained group without LuG

Figure 5 shows the results of flow cytometry tests. The data indicate that most HepG2 cells remained in the normal phase across all treatment concentrations of luteolin-7-*O*-glucoside (5, 30, 50 µg/ mL) in treatment groups (d, e, f) compared to Annexin group (a) and PI group (c). Furthermore, the treatment groups exhibited a cell cycle distribution similar to that of the auto group (b) and the unstained group without LuG (g). Based on the flow cytometry results, it can be concluded that the cytotoxicity of luteolin-7-*O*-glucoside on HepG2 cells did not involve necrotic or apoptotic pathways. The cell cycle test was then performed. In contrast to previous studies, luteolin-7-*O*-glucoside (LuG) in this study induced a non-apoptotic form of cell death. This suggested involvement of alternative regulated cell death pathways, such as ROS-mediated autophagy or proliferative arrest. Flavonoids often activate this pathway by modulating signaling cascades such as AMPK, mTOR, PI3K/Akt, Beclin-1, and LC3, resulting in increased autophagosome formation and degradation of cellular component.^16^. For example, Kaempferol promotes autophagic cell death in HCC lines (including HepG2) via AMPK activation, ULK1 phosphorylation, mTORC1 inhibition, increased Beclin-1, LC3-II, and decreased p62 levels.^17^ Furthermore, Oxeiptosis is a form of programmed, non-inflammatory, caspase-independent cell death activated under high oxidative stress. It involves the KEAP1/PGAM5/AIFM1 signaling axis, where elevated ROS disassemble the KEAP1–PGAM5 complex, leading PGAM5 to dephosphorylate AIFM1. AIFM1 then remains mitochondrial. Auriculasin (a member of isoflavanoids has been reported in *Maclura pomifera*) induces oxeiptosis, apoptosis, and ferroptosis depending on context, enhancing effectiveness against cancer cells.^18^. Also, in 2022, Yang *et al*. demonstrated quercetin, as a member of the flavonoid group, has been implicated in inducing non-apoptotic modes including mitotic catastrophe, senescence, and ferroptosis in various cancer models.^19^

###  Cell Cycle Analysis

**Figure 6:**
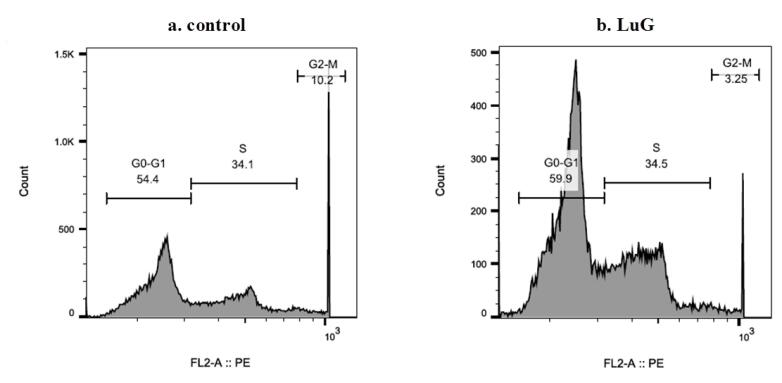
Effect of Luteolin-7-*O*-glucoside on cell cycle progression in HepG2 cells. **a.** HepG2 cells were incubated at 37 °C. **b.** HepG2 cells were incubated at 37 °C with Luteolin-7-*O*-glucoside for 24 h

 As illustrated in Figure 6, the analysis of the cell cycle revealed that the 2N DNA content (G0/G1 phase) slightly increased by approximately 5%. In contrast, no significant changes were observed in the S phase, which represents the synthesizing DNA phase. However, a decrease of about 7% was noted in the 4N DNA content (G2/M phase), indicating that fewer cells were progressing to mitosis. Following incubation, the cells were harvested, fixed, and stained with propidium iodide. The stained cells were then analyzed using flow cytometry to assess their distribution across the different phases of the cell cycle. This analysis provided insights into the effects of luteolin-7-*O*-glucoside on cell cycle progression in HepG2 cells.

 Luteolin, with a flavonoid structure, and its derivatives have been shown to have anticancer effects in various cancer cell lines.^20,21^ luteolin was synthesized through a double bond addition between carbon-2 and carbon-3 in the C-ring of eriodictyol, and hydroxyl group addition to apigenin by the flavonoid-3′-hydroxylase enzyme is another metabolic pathway for the synthesis of luteolin.^22^ Luteolin, when administered orally, is absorbed in the small intestine and subsequently metabolized in the liver through glucuronidation and sulfation pathways. Ultimately, it is excreted from the body via feces and urine.^23-25^ This pattern confirmed the pharmacokinetics of luteolin, showing that the aglycone form of flavonoids accumulates in the liver and gastrointestinal tissue.^26^ Additionally, El-Sharawy *et al*. reported that 99mTc-luteolin was eliminated via the kidney and fecal routes in mice.^27^ The radiolabeling of luteolin and its derivative results showed that these compounds avoided first-pass metabolism and were metabolized directly in the liver, where they first accumulated. As time passed, the percentage of accumulation in the liver tissue decreased, while this accumulation increased in other organs, such as the stomach, intestine, kidney, and bladder. Due to the presence of glucose in luteolin-7-*O*-glucoside and the higher hydrophilicity of this compound, the elimination phase of the glucoside form of luteolin appeared more rapidly than that of its aglycone form. Lin *et al*. demonstrated that Luteolin-7-*O*-glucoside is hydrolyzed to luteolin in the gastrointestinal tract.^28^ However, ^99m^Tc-Lu was initially distributed throughout the body (due to the absence of sugar in its structure) and then accumulated in the liver and other targeted organs.

 Due to their high accumulation, the HepG2 and SW480 cancer cell lines were chosen for further study. However, no inhibitory effects were observed on the SW480 cell line. In fact, other studies have indicated that luteolin can inhibit the proliferation and migration of colon cancer cells, potentially through the IL-6/STAT3 signaling pathway.^29^ Additionally, Lu and LuG did not demonstrate any cytotoxicity on normal cells (HUVEC cell line). Witkowska-Banaszczak *et al*. reported that luteolin-7-*O*-glucoside has a dose-dependent effect on the viability of the HepG2 cell line.^30^ The aglycone form of luteolin was found to be less effective against HepG2 cells compared to the glycone form, which may be due to differences in cellular uptake and metabolism. However, other studies have demonstrated that both luteolin and its derivatives can suppress cancer cell growth without adversely affecting normal cells. For instance, Chen *et al*. reported no cytotoxicity of luteolin-7-*O*-glucoside (LuG) on normal Huh7 liver cells.^31^ Many studies have indicated that the anticancer effects of luteolin are mediated via apoptosis. Lee *et al*. demonstrated that luteolin triggers classical mitochondrial apoptosis in HepG2 (Bax/Bak translocation, cytochrome C release, caspase-3 activation).^32^ Flow cytometry for apoptosis/necrosis (Annexin V/PI) showed no significant increase in early apoptosis (Annexin V + /PI–) or late apoptosis/necrosis (Annexin V + /PI + ) compared to controls on HepG2 cells after 24 h with LuG treatment. At the same time, Chen *et al*. reported that Luteolin-7-*O*-glucoside induced apoptotic features in HepG2 cells.^31^ The mentioned study indicates that an Annexin signal should have been observed in the present study. However, this discrepancy may be attributed to differences in dosage or time-course. Additionally, Chen *et al*. demonstrated that luteolin glycoside has caspase-independent activity, as evidenced by increased cleaved PARP without caspase activation. In contrast, Lee *et al*. showed caspase-3 cleavage with luteolin.^31,32^ This study demonstrated cell death induced via a non-apoptotic mechanism by glycosylated luteolin. Furthermore, other flavonoids such as quercetin and apigenin yield strong Annexin/PI signals.^33^

 Cell cycle analysis of LuG-treated HepG2 cells revealed impaired G1 to S progression, resulting in fewer cells reaching the G2/M phase. In contrast, Chen *et al*. reported that 24 h of LuG treatment induced G2/M phase in HepG2.^31^ Overall, flavonoids can arrest cancer cells at various checkpoints. Previous studies have shown that luteolin and quercetin inhibit the G1 phase by downregulating cyclin D1 and p21, as well as the G2/M phase by decreasing cyclin B1 and CDC2 levels.^33,34^ But, apigenin induces G0/G1 phase by inhibiting the phosphorylation of the Rb protein.^35^ While some literature indicates that flavonoids induce G1 and/or G2/M phase, this study found that LuG-treated HepG2 cells exhibited cell cycle inhibition. This divergence may be related to variations in cell type, concentration, and treatment duration. The difference between this study and previous studies may be due to the natural source of luteolin. Both isomeric forms of glucose exist in the plants. α-Glucose and β-glucose are stereoisomers, and their pharmacokinetic and pharmacodynamic properties differ. In addition, the cytotoxicity effects of flavonoids are time- and dose-dependent. In other words, different times and doses can induce and activate different apoptotic and non-apoptotic death cell pathways.

 In summary, the findings of the present study align with the established characteristics of the glycoside form of luteolin, including its vigorous anti-proliferative activity in HepG2 cells, predominant hepatic distribution, and ability to induce cell cycle arrest. The inhibition of cell cycle progression in the S-phase and depletion from the G2/M phase is typical of DNA-targeting drugs, such as antimetabolites (e.g., 5-FU and gemcitabine) or replication inhibitors.

## Conclusion

 The biodistribution and cytotoxicity studies of luteolin-7-*O*-glucoside indicate that this flavonoid preferentially accumulates in liver tissues and effectively inhibits HepG2 cell viability. However, the hypothesis suggesting a relationship between the target organ and the effectiveness on cancer cells in that organ was rejected, particularly concerning the colon cell line (SW480). In other words, tissue accumulation alone did not ensure efficacy outside the liver. These observations demonstrate a clear tissue-selective pharmacological profile with vigorous anticancer activity on hepatic cancer cells and no cytotoxic effects on non-hepatic cells. The liver biodistribution of luteolin-7-*O*-glucoside may be responsible for its anti-HepG2 activity. Therapeutically, the strong liver affinity of luteolin-7-*O*-glucoside could be leveraged to treat hepatocellular carcinoma more effectively, potentially allowing for high local efficacy with reduced off-target toxicity.

## Competing Interests

 Authors declare no conflict of interest.

## Ethical Approval

 This study was approved in 2019 by the Ethics Committee of Tehran University of Medical Sciences, Tehran, Iran (IR.TUMS.TIPS.REC.1398.040).
